# Nasal endoscopy and localization of the bleeding source in epistaxis: last decade's revolution

**DOI:** 10.1016/S1808-8694(15)31302-1

**Published:** 2015-10-20

**Authors:** Glauco Soares de Almeida, Camilo A. Diógenes, Sebastião D. Pinheiro

**Affiliations:** 1 Specialist in Otorhinolaryngology, Physician with Hospital Otoclínica; 2 Generalist (Emergency physician); 3 Joint Professor, Medical School, Federal University of Ceará, Head of the Service of Otorhinolaryngology. Hospital Otoclínica - Fortaleza, Ceará

**Keywords:** epistaxis, nasal bleeding, treatment

## Abstract

**Summary:**

Epistaxis remains one of the most common otolaryngology emergencies. Despite considerable interest in the subject, there is still no consensus on the most appropriate primary therapeutic modality.

**Aim:**

The purpose of this study was to evaluate the bleeding source of acute or recurrent epistaxis in adults.

**Study design:**

Clinical prospective.

**Material and Method:**

Thirty adults patients with acute or recurrent epistaxis were evaluated through the use of frontal light and endoscope for identification of the bleeding source in the nasal cavity.

**Results:**

Use of the nasal endoscope allowed diagnosis of the bleeding site in all patients.

**Conclusion:**

A careful examination of the posterior nasal cavity allows identification of the bleeding source in most patients and should be a routine procedure.

## INTRODUCTION

Not so many issues in Otolaryngology have so deeply shifted their paradigms in the last decades as the treatment of epistaxis. Terms like “untreatable epistaxis” and “conservative treatment” should be revised. The main reason for this paradigm shift was the advent of nasal endoscopy. If epistaxis was previously labeled as an “untreatable” condition and managed with multiple nasal packing, ligature of the carotid and/or maxillary arteries^1^, ^2^, or even vessel embolization^3^, now they have shown to be easily diagnosed and treated through nasosinusal endoscopic surgery. Treatments previously considered “conservative”, such as nasal packing seem much more traumatic, uncomfortable and, in some cases, with higher risks^4^ than simple endoscopic procedures, such as local cauterization or ligature of the sphenopalatine artery.

For effective therapeutic approach, it is crucial that nasal vascularization and prevalent bleeding sources are better understood. Ligature of the external carotid conducted by Hyde^2^ in 1935 was the first vascular procedure for epistaxis control. Chandler^1^, in 1965 was the first to perform a ligature of the maxillary artery transantrally in an attempt to intervene next to an intranasal bleeding site. Intranasal approaches for epistaxis control were established after the first ligature of the sphenopalatine artery using a microscope (Stamm, 1985)^5^ and an endoscope (Budrovich and Saette, 1992)^6^. Since then, treatment of epistaxis under microscopic or endoscopic magnification of the nasal cavity posterior segment became popular, less threatening and reduced distress.

## OBJECTIVE

This study aims at identifying the nasal cavity's bleeding source of patients with active or recurrent epistaxis by means of nasal videoendoscopy.

## MATERIAL AND METHOD

A prospective study was conducted with 30 patients with epistaxis who were assisted at the emergency otolaryngology service (Otoclinica - Fortaleza, CE), in the period of January 2002 to August 2004. Ages ranged from 32 to 68 years, with mean age of 52. The group of patients comprised 17 men (56.6%) and 13 women (43.3%). After clinical assessment, all patients were initially examined by classical anterior rhinoscopy with frontal illumination, while those whose bleeding sources were not identified were submitted to nasal endoscopic evaluation. Bleeding sources were classified as: anterior or posterior; from the lateral nasal wall or nasal septum.

## RESULTS

Out of 30 patients assessed, 19 (63.2%) presented bleeding in the posterior segment of the nasal cavity – 14 (46.6%) in the nasal septum and 5 (16.6%) in the lateral nasal wall. Out of 11 patients (36.6%) with bleeding at the anterior segment of the nasal cavity, all bleedings were found in the anterior nasal septum. No patients presented bleeding in the anterior region of the lateral nasal wall or bilateral bleeding.

## DISCUSSION

W. Messerklinger^7^ was the first to adopt nasal and paranasal endoscopic surgery, rendering further contributions to otolaryngologists. Since 1985, this approach started to be broadly practiced by Kennedy^8^ in the United States, and worldwide in the 90's.

In 1992, when Budrovich^6^ reported the treatment of epistaxis by nasal endoscopy, several other studies were published. The first articles on this technique for the control of epistaxis described comprehensive maxillary antrostomy followed by removal of the posterior wall of the maxillary sinus and ligature of pterygomaxillary fossa vessels (White, 1996). The improved approach was very radical concerning nasal vascularization, although it did not play a direct effect over the intranasal bleeding source. After various studies on anatomy micro-dissections of cadavers, ligatures of sphenopalatine vessels to reach the nasal cavity^9^, ^10^, ^11^, ^12^, ^13^ were the following step. The concept that vascular ligature is more effective when performed the nearest possible to the bleeding source led nasal endoscopy to become the gold standard approach for patients with epistaxis.

Regular use of clinical endoscopy during the last decade amplified the knowledge on the etiology and treatment of epistaxis. The bleeding source inside the nasal cavity could be more easily and accurately identified. Moreover, other less invasive procedures, such as cauterization of the bleeding source, could be done presenting high efficacy rates^14^. Local cauterization of the bleeding spot, which was previously limited to anterior portions of the nasal cavity, could be amplified to posterior regions, with the advent of endoscopic visualization.

Clinical use of endoscopy showed that, except for the anterior nasal septum as a bleeding source, the most frequent site of epistaxis was the posterior portion and not the lateral wall of the nasal septum, which is contrary to what was previously believed, but corroborates our casuistic and the data in the literature available^14^, ^15^. The literature also emphasizes the importance of Woodruff's venous plexus, which corresponds to less than 10% of the cases with posterior epistaxis.

These clinical observations corroborate reports of highly effective cauterization of the sphenopalatine artery (and/or its branches: posterior lateral and septum nasal artery) in the control of posterior epistaxis, although it opens new possibilities for less invasive approaches by local cauterization of the bleeding spot in the posterior nasal septum through endoscopic visualization, which also presents high efficacy rates^16^, ^17^.

## CLOSING REMARKS

The nasal septum is the most frequent site for posterior nasal bleeding. If the bleeding source is not identified by anterior rhinoscopy, a nasal endoscopy is mandatory. Identification and cauterization of the bleeding point under endoscopic magnification of the posterior nasal septum becomes an effective and less invasive procedure, avoiding unnecessary cauterization of the sphenopalatine artery.
